# Electrochemical Biosensors for Express Analysis of the Integral Toxicity of Polymer Materials

**DOI:** 10.3390/bios13121011

**Published:** 2023-12-04

**Authors:** Natalia Yu. Yudina, Tatyana N. Kozlova, Daniil A. Bogachikhin, Maria M. Kosarenina, Vyacheslav A. Arlyapov, Sergey V. Alferov

**Affiliations:** 1 Chemistry Department, Federal State Budgetary Educational Establishment of Higher Education, Tula State University, 300012 Tula, Russia; tysia21-05-90@mail.ru (N.Y.Y.); v.a.arlyapov@tsu.tula.ru (V.A.A.); 2 Laboratory of Ecological and Medical Biotechnology, Tula State University, Friedrich Engels Street 157, 300012 Tula, Russia; kozlovatatyana_1993@mail.ru (T.N.K.);

**Keywords:** toxicity, microbial biosensor, EC_50_, toxicity index (T), bacteria *Gluconobacter oxydans*

## Abstract

Biosensors based on an oxygen electrode, a mediator electrode, and a mediator microbial biofuel cell (MFC) using the bacteria *Gluconobacter oxydans* B-1280 were formed and tested to determine the integral toxicity. *G. oxydans* bacteria exhibited high sensitivity to the toxic effects of phenol, 2,4-dinitrophenol, salicylic and trichloroacetic acid, and a number of heavy metal ions. The system “*G. oxydans* bacteria–ferrocene–graphite-paste electrode” was superior in sensitivity to biosensors formed using an oxygen electrode and MFC, in particular regarding heavy metal ions (EC_50_ of Cr^3+^, Mn^2+,^ and Cd^2+^ was 0.8 mg/dm^3^, 0.3 mg/dm^3^ and 1.6 mg/dm^3^, respectively). It was determined that the period of stable functioning of electrochemical systems during measurements was reduced by half (from 30 to 15 days) due to changes in the enzyme system of microbial cells when exposed to toxicants. Samples of the products made from polymeric materials were analyzed using developed biosensor systems and standard biotesting methods based on inhibiting the growth of duckweed *Lemna minor*, reducing the motility of bull sperm, and quenching the luminescence of the commercial test system “Ecolum”. The developed bioelectrocatalytic systems were comparable in sensitivity to commercial biosensors, which made it possible to correlate the results and identify, by all methods, a highly toxic sample containing diphenylmethane-4,4′-diisocyanate according to GC-MS data.

## 1. Introduction

Increasing production rates and the emergence of new materials are leading to an increase in the demand for early testing of products for the presence of substances that cause a cascade of toxic effects that lead to human health problems and a decrease in biodiversity [1,2,3]. Among the main toxicants in polymer materials are the organic compounds used, such as plasticizers, fillers, and dyes [4], and heavy metals that can accumulate in the human body through bioaccumulation [5].

Various physicochemical methods are used to detect individual toxicants [6,7]. However, it is advisable to determine the integral toxicity index in the test sample based on the reactions of living organisms to assess the synergistic effects of toxicants and identify the toxic effects of unknown compounds that are decomposition products. Often, poorly reproducible results and the duration of standard analysis methods that use biological test systems (ciliates, daphnia, fish, algae, and duckweed) do not allow for the prompt and reliable determination of the toxicity of the studied samples.

In the last decade, biosensor systems, that include a biological recognition element and a physicochemical transducer, have been actively developed to detect pollutants due to their sensitivity, selectivity, portability, and the possibility of miniaturization [8,9]. The similarity of the physiological reactions and cellular organization of microorganisms with higher organisms that are exposed to chemical contamination allows us to obtain more accurate and reliable results. There are a number of commercial biosensors for assessing toxicity based on bioluminescent bacteria (Microtox (AzurEnvironmental, Carlsbad, CA, USA), LUMIStox (Beckman Instruments, High Wycombe, UK), Tox-Alert (Merck, Rahway, NJ, USA), ToxScreen (ChekLight Ltd., China), and Biotox-10M (Nera-S, Moscow, Russia)) [10]. Since these devices are based on quenching the bioluminescence of bacteria (*Aliivibrio fischeri* (formerly *Photobacterium phosphoreum*), *Photobacterium leiognathi*, *Vibrio qinhaiensis*, and genetically engineered *Escherichia coli*), their use is limited for the analysis of solutions of increased turbidity and color, with non-optimal pH and temperature [10,11]. In addition, the genetically modified microorganisms used in them are quite expensive.

Therefore, microbial electrochemical biosensors of various designs (including biofuel cells (BFC), biosensors based on the analysis of oxygen consumption, or the use of artificial electron acceptors) are attracting more attention. The selection of electrode material and bioreceptor system can lead to the development of highly sensitive and selective detection of individual toxicants being analyzed, or the overall toxic reaction of the test object can be assessed by reducing oxidative activity. In recent years, review and experimental articles present whole-cell biosensor systems of various types of detection for the determination of highly toxic heavy metals (lead, mercury, cadmium), phenolic, and organophosphorus compounds [12,13].

The design features of the biosensor are of decisive importance when developing biosensors for assessing toxicity. The amperometric biosystem is the easiest to operate and cheapest for determining toxicity. This system is based on the Clark oxygen electrode with microbial cells immobilized on the surface [14,15]. Due to these advantages, such a system can be easily standardized, validated, and implemented in analytical laboratories for daily routine analysis. The use of mediators in second-generation biosensors makes it possible to significantly increase the current value by facilitating electron transfer from the biomaterial to the electrode and thereby improve the sensitivity of electrochemical biosensors in detecting toxic compounds such as phenols [16,17] and heavy metal ions (Pb^2+^, Cd^2+^, Cu^2+^, and Zn^2+^) [16,17,18]. A potential alternative for detecting various toxicants is microbial biofuel cells (MFCs), in which the output signal (voltage and current) depends on changes in environmental conditions for microorganisms in the anode chamber. Such systems can be effectively used for the detection of heavy metals [19], organic substances [20] and antibiotics [21]. However, integral toxicity analysis using MFC remains problematic due to the problem of low sensitivity compared to mediator microbial biosensors, which is associated with high internal electrical resistance [22]. Therefore, depending on the electrode material, membrane type, operating mode, and configuration of biosensor cells, the efficiency of determining integral toxicity will vary significantly.

**Figure 1:**
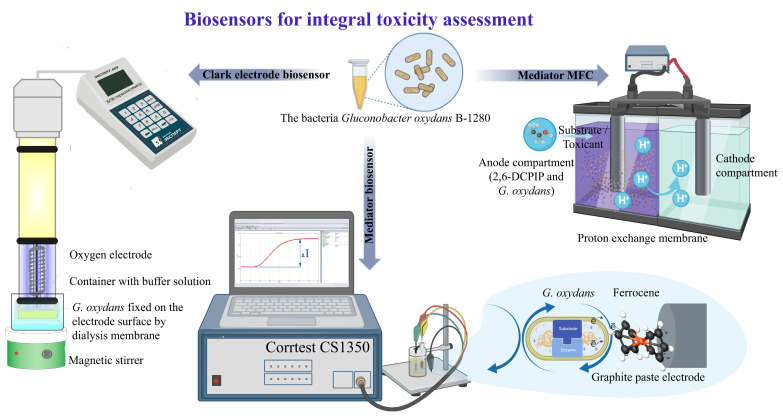
Study of the toxicity of polymeric materials using different approaches.

The purpose of this work was to compare the sensitivity and stability of biosensors based on the bacteria *G. oxydans* in the composition of an oxygen electrode, an MFC, and a mediator-type electrode, intended to determine the integral toxicity of polymer materials (Figure 1). The bacteria *Gluconobacter oxydans* BKM-1280 was used as a test object in the presented work for the development of microbial biosensors. This microorganism is characterized by the structural features of the bacterial cell and its metabolism, determined by the periplasmic orientation of the active centers of membrane-bound enzymes [23], which ensures accessibility to substrates.

Since the current scientific literature does not describe a study on the efficiency of integral toxicity determination using electrochemical biosensors with different types of signal registration based on a single microbial strain as a biorecognizing element, this experimental work may be useful for the design of more stable and promising biosystems for toxicity analysis using microorganisms. 

## 2. Materials and Methods

### 2.1. Reagents and Materials

All chemicals were of analytical grade and used without further purification. To prepare the solutions, deionized water prepared using the Aqualab AL-1 Double system (Aqualab LLC, Moscow, Russia) was used. Yeast extract, agar-agar, trichloroacetic (TCA) and salicylic acids, inorganic salts (Dia-M, Moscow, Russia), D-sorbitol, phenol, 2,4-dinitrophenol (HIMMED, Moscow, Russia), D-glucose, acetonitrile (Scharlab, Barcelona, Spain), ferrocene, sodium 2,6-dichlorophenolindophenolate (2,6-DCPIP), cellulose dialysis membrane D9777 (pore size 12 kDa) (Sigma-Aldrich Chemicals, Darmstadt, Germany), and graphite powder and mineral oil (Fluka, Bush, Swizerland) to form MFCs, graphite rods (NIIEI, Russia) and a proton-selective membrane MF-4SK (Plastopolimer, St-Petersburg, Russia) were used.

### 2.2. Cultivation of Microorganisms

The strain *Gluconobacter oxydans* VKM B-1280 was provided by the All-Russian Collection of Microorganisms from the Institute of Biochemistry and Physiology of Microorganisms of the Russian Academy of Sciences (Pushchino, Russia). *G. oxydans* bacteria were cultivated on agar containing D-sorbitol (200 g/dm^3^), yeast extract (20 g/dm^3^), and agar-agar (20 g/dm^3^), and were subcultured monthly. An inoculum of *G. oxydans* cells was obtained by aerobic cultivation at 28 °C for 24 h in test tubes filled with 15 mL of medium consisting of D-sorbitol (200 g/dm^3^) and yeast extract (20 g/dm^3^) on a BIOSAN ES-20/60 incubator shaker (BioSan, Rīga, Latvia). The cell biomass was grown in 150 mL of the liquid medium in shaking flasks with the resulting inoculum until reaching the late exponential phase (18 h) when the cells contained the most active pyrroloquinoline quinone-dependent dehydrogenases with the highest yield. After cultivation, the cells were collected by centrifugation for 10 min at 8000 rpm (MPW MEDINSTRUMENTS 04-347, Warsaw, Poland) and washed twice with 30 mM of a Na-phosphate buffer with a pH of 6.0. The settled cells were resuspended in a new portion of the buffer, distributed into Eppendorf microtubes, and centrifuged for 10 min at 12,000 rpm (centrifuge MPW MEDINSTRUMENTS 04-347, Warsaw, Poland). The resulting cell sediment was air-dried for an hour and frozen for long-term storage at −18 °C.

### 2.3. Formation of Working Electrodes

To form a biorecognition element of a biosensor based on an oxygen electrode, 10 μL of a suspension of bacteria diluted in a 1:1 ratio with 30 mM of Na-phosphate buffer solution with a pH of 6.0 was applied to a fragment of a dialysis membrane measuring 1 × 1 cm (the titer of *G. oxydans* bacteria is 2.3 × 10^8^ CFU/mL). The biorecognition element was fixed to the electrode using a polymer ring.

To form a mediator biosensor, 10 μL of a bacterial suspension (*G. oxydans* bacterial titer, 4.5 × 10^8^ CFU/mL), obtained as described above, was applied to previously prepared working electrodes. To prepare the working electrode, 90 mg of graphite powder, 10 mg of ferrocene, 40 μL of mineral oil, and 500 μL of acetone were stirred until the acetone evaporated and the resulting mixture was filled into a plastic tube. The formed working electrodes were left to air dry for 15 min, after which the surface of the electrode was covered with a dialysis membrane, which was secured with a plastic ring, to fix the biomaterial and prevent it from being washed out.

In experiments using MFC as a biocatalyst, bacterial cells were used in the anode space (*G. oxydans* bacteria titer −8.6 × 10^8^ CFU/g wet biomass), which were stored at room temperature before measurements in the form of a suspension at a concentration of 300 mg/cm^3^ in 30 mM Na-phosphate buffer pH 6.0.

### 2.4. Electrochemical Measurements

**Figure 2:**
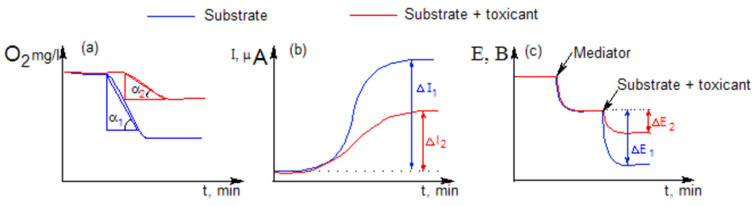
Schemes for recording responses of different types of biosensors: (**a**) biosensor based on oxygen electrode; (**b**) mediator type biosensor; (**c**) MFC based biosensor.

Biosensor measurements on a Clark-type oxygen electrode (DKTP-02) with immobilized bacteria were carried out using the EXPERT-001 analyzer (Econix-Expert, Moscow, Russia) interfaced with a personal computer running specialized software EXP2PR version from V16.10.09 (Econix-Expert, Moscow, Russia). A substrate (glucose solution 1 mol/dm^3^) was added to a measuring cell with a volume of 5 cm^3^ with constant stirring with a magnetic stirrer (250 rpm) to 4 cm^3^ of Na-phosphate buffer with a pH of 6.0. A decrease in oxygen concentration in the near-electrode space was recorded with a measuring sensor as a result of an enzymatic reaction. The biosensor signal was the maximum rate of change in oxygen concentration upon the addition of substrates (mgO_2_/(dm^3^ × s)) (Figure 2a).

An electrochemical station “CORRTEST” (Corrtest Instruments, Wuhan, China) was used to carry out measurements on the mediator biosensor and MFC. In the case of a mediator biosensor, the electrochemical signal was recorded using a two-electrode system consisting of an Ag/AgCl electrode (reference electrode) and a working graphite-paste electrode. The measurements were carried out at a constant potential of 0.25 V relative to the silver chloride electrode. After establishing a stationary current value, an aliquot of the substrate (glucose solution 1 mol/dm^3^) was added to the cuvette (volume 5 cm^3^) and waited for the next stationary state. The amplitude of the change in current strength before and after introducing the substrate into the measuring cell (∆I, μA) was taken as the response of the biosensor (Figure 2b).

The biofuel cell consisted of anode and cathode chambers of the same volume (5 cm^3^), separated by a proton-selective membrane. Spectral graphite rods with a diameter of 8 mm were used as electrodes (immersion depth 10 mm). The electrodes were washed until the potential value was 0 mV. A suspension of bacteria (3 mg/mL) and 2,6-DCPIP (concentration 150 μM) was added with constant stirring with a magnetic stirrer (400 rpm) into the anode compartment to a working solution with a volume of 3 cm^3^ (30 mM Na-phosphate buffer solution with a pH of 6.0). After the stationary potential value was established, a glucose solution (concentration in the anode chamber 10 mM) was introduced into the anode chamber. In this way, the response to the addition of the substrate was recorded as the amplitude of the generated potential difference (∆E, mV) (Figure 2c).

### 2.5. Sampling and Sample Preparation for Biotesting Methods

A total of six samples of consumer goods made of polymer materials were studied. Of these samples, three suggested contact with food products (water bottle, food container, baby bottle with pacifier), and three suggested contact with human skin (phone case, dousing gloves, medical gloves). Sample preparation for biotesting methods involved the preparation of aqueous extracts of the materials under study. All samples were cut into pieces measuring 2 mm × 2 mm with a thickness of no more than 5 mm. A crushed sample of products (1 ± 0.01 g) was placed in a vessel with a ground-in stopper, filled with a 50-fold volume (50 cm^3^) of distilled water (pH 6.8–7.4), mixed thoroughly, ensuring complete wetting of the sample with water, and thermostated at 40 °C for 24 h (dry air thermostat TV-80-1, Kasimovsky Instrument Plant, Kasimov, Russia).

### 2.6. Extraction of Samples for Chromatography

The crushed sample material was placed in glass containers (grinding was carried out as described above). 20 cm^3^ of acetonitrile was added to the samples and sonicated in an ultrasonic bath (Guangzhou Hanker Electronic Technology Co., Ltd., Guangzhou, China) for 1 h at room temperature according to the method [24] to extract toxic substances. Acetonitrile was transferred into clean glass vials and immediately used for analysis.

### 2.7. Chromatography Analysis of Samples

Extracts in acetonitrile were analyzed using a Kristall-4000M gas chromatograph with a Maestro-AMS mass detector (MSD) (injection volume 1 μL) on a ZB-5ms column (length 30 m and Ø 0.25 mm, stationary phase −5% phenyl-arylene 95% methylpolysiloxane, phase thickness 0.25 µm). The chromatography conditions were as follows: a carrier gas (helium; total flow 80.6 cm^3^/min), an initial thermostat temperature of −60 °C, a final thermostat temperature of −300 °C, a temperature increase rate of −20 °C/min, an evaporator temperature of −300 °C, an MSD time range from 2.5 to 15 min, and a solvent pass of 2.5 min. The identification of compounds was carried out by comparison of mass spectra with the NIST 14 mainlib/replib library (score ≥ 60%).

### 2.8. Determination of Toxicity Using the “Ekolum” Test System

Rehydration of the lyophilized bacterial test system “Ecolum”, control of the error of the toxicological analysis technique, and the biotesting procedure using the Biotox-10M device were carried out according to the method attached to the device. For each sample, three control–experiment pairs were measured sequentially. The toxicity of the sample was assessed by the relative difference in the intensity of bioluminescence of the control and experimental samples and the calculation of the toxicity index (T) using the formula:

(1) $$T=\frac{I_{K}-I_{0}}{I_{K}}\times 100\%,$$

where I_K_ is the luminescence intensity of the control sample of bacteria, and I_0_ is the luminescence intensity of bacteria after adding an aqueous extract of the test sample.

### 2.9. Standard Biotest Method Based on the Test Object L. minor

The toxicity of aqueous extracts of the studied polymeric materials was assessed by the inhibition of the yield of duckweed (*L. minor*), expressed as a percentage (I_y_) [25]. Containers with control and experimental samples were kept for 7 days under a fluorescent lamp. The average percent yield inhibition was calculated as follows (2):

(2) $$I_{y}=\frac{b_{C}-b_{T}}{b_{C}}\times 100\%,$$

where I_y_ is the percentage of yield inhibition, b_C_ is the final biomass in the control, and b_T_ is the final biomass in the experimental sample.

If the inhibition level was 20% or more, the sample was considered toxic.

### 2.10. Bioassay Using Bovine Sperm

The safety assessment of the products made from polymer and textile materials was carried out using cattle sperm as a test object [26]. The mobility index (I_t_) was measured using an AT-05 image analyzer (BMK-INVEST, Kaluga, Russia). A sample was considered toxic if the obtained index value was not within the interval 70% < I_t_ < 120%.

## 3. Results and Discussion

### 3.1. Development of Electrochemical Biosensors with Different Types of Signal Recording for Assessing Integral Toxicity

The bacteria *G. oxydans* was chosen as an effective biocatalyst. Previously, *G. oxydans* was successfully used for environmental monitoring in bioelectrochemical systems of all types (based on oxygen electrodes [14], mediator type [27,28], and MFC [29,30,31]). Earlier, during the development of a biosensor based on an oxygen electrode to assess integral toxicity, the sensitivity of bacteria to both the toxic effects of organic pollutants and heavy metals was established [14].

The use of different bioelectrochemical systems (using an oxygen electrode, a mediator graphite-paste electrode, and MFC) will identify the most sensitive test system for assessing toxicity, since in these three systems the reaction at the electrode is caused by different biochemical pathways of electron transfer from the bacteria *G. oxydans*. In the presence of a toxicant in a microbial cell, some functions of cellular metabolism are disrupted, and the rate of oxygen consumption during substrate oxidation decreases, which serves as an indicator of inhibition in a system based on an oxygen electrode. In mediator systems, the final acceptor of electrons in the microbial respiratory chain is redox-active substances (mediators) that remove electrons at different stages of the electron transfer chain (ETC), which leads to a change in the test reaction of bacteria to the same toxicant. Ferrocene [16] and 2,6-DCPIP [31] were used, respectively, to obtain a biosensor signal (Figure 2) independent of oxygen partial pressure and improve the efficiency of electron transport from the bacterial cell to the electrode surface in the mediator biosensor and MFC.

The difference in the generated current/potential in the systems “immobilized bacteria–oxygen electrode”, “immobilized bacteria–ferrocene–graphite-paste electrode” and “suspension of bacteria–2,6-DCPIP–graphite electrode” as a result of oxidation of the substrate by the enzymatic system of bacteria in the presence and in the absence of toxicants in the analyzed sample will be the inhibition value (toxicity index T, %) (3):

(3) $$T=\frac{R_{substrate}-R_{substrate+toxicant}}{R_{substrate}}\times 100\%,$$

where R_substrate_ is an electrochemical signal for the introduction of a substrate into the system in the absence of a toxicant, and R_substrate+toxicant_ is an electrochemical signal for the introduction of a substrate into the system in the presence of a toxicant.

**Figure 3:**
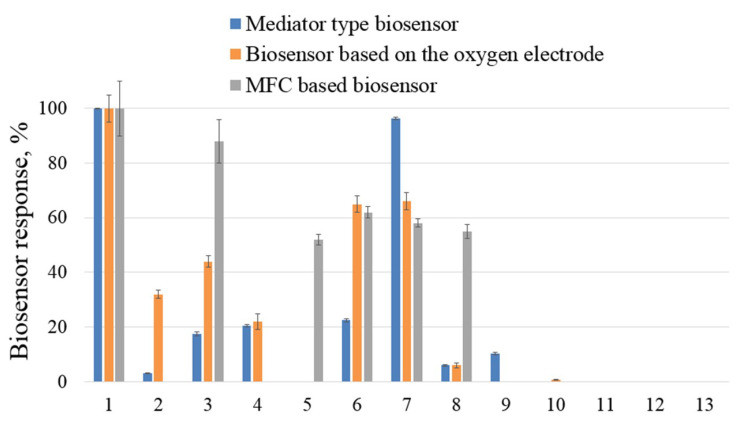
Substrates oxidized by bacteria *G. oxydans*: 1—glucose; 2—fructose; 3—galactose; 4—sucrose; 5—methanol; 6—ethanol; 7—butanol-1; 8—glycerin; 9—formaldehyde; 10—phenol; 11—2,4-dinitrophenol; 12—salicylic acid; 13—TCA (*n* = 3, *p* = 0.95). Data on biosensor based on the oxygen electrode from [14], data on MFC based biosensor from [32].

The range of oxidized substrates by *G. oxydans* bacteria was assessed as part of biosensors based on an oxygen electrode (substrates no. 11–13) [14], MFC [32] and a mediator type to identify substances that may have a negative effect. In this work, the oxidative activity of microorganisms was additionally studied by adding substrates No 11–13 (oxygen electrode) and No 9–13 (MFC) to the measuring cell. Data on the range of substrates oxidized by *G. oxydans* bacteria are presented in percentages relative to the maximum response of the biosensor to glucose in the diagram (Figure 3).

*G. oxydans* bacteria do not oxidize phenol, 4-nitrophenol, 2,4-dinitrophenol, TCA, and salicylic acid; therefore, these substances can be selected as model toxicants. For the biosensor based on Clark’s electrode, the absence of responses to formaldehyde was recorded, and in the mediator biosensor, to phenol. This change in the range of oxidizable substrates of the bacteria *G. oxydans* in different types of biosensors is explained by the peculiarities of the reduction of the mediator during its interaction with the ETC of microorganisms.

The toxic effect of substances that are not subject to metabolic transformation by *G. oxydans* bacteria was assessed by a decrease in the analytical signal for glucose, as the most intensively oxidized substrate. The low probability of the presence of glucose in the composition of the studied samples of polymeric materials will not introduce an analytical error when determining their toxic effect on the conformation of the active center of glucose oxidase, which is characterized by absolute specificity.

### 3.2. Main Characteristics of Biosensors for Determining Toxicity

The stability (in the absence and under the influence of a toxicant (Zn^2+^, EC_50_)) and sensitivity of the electrochemical system were studied to assess the possibility of using a biosensor with a certain type of signal recording based on the bacteria *G. oxydans* to determine integral toxicity. The dependences of the biosensor response to glucose solution concentrations were approximated by Equation (4) since the electrochemical response was provided by the enzymatic reactions of microorganisms within Michaelis-Menten kinetics:

(4) $$V=\frac{V_{max}\times [S_{0}]}{K^{\prime }+[S_{0}]},$$

where V is the biosensor response, V_max_ is the maximal enzymatic reaction rate reached at [S] → ∞ (maximum response of the biosensor), and K′ is the apparent Michaelis constant, i.e., the substrate concentration, at V = V_max_/2.

The study on the influence of the pollutant on the operational and long-term stability of the sensors was carried out in the presence of Zn^2+^. This toxicant has a negative effect on the oxidative activity of microorganisms in all types of biosensors and is used as a reference for monitoring the error of the toxicological analysis method based on the “Ecolum” bacterial test. When the oxidative activity of bacteria decreased by 50% of the maximum, the biosensor was considered unsuitable for measurements. Table 1 presents the main characteristics of biosensors based on the bacteria *G. oxydans* for toxicity assessment.

Electrochemical systems are designed to establish the duration of stable operation in the absence of exposure to pollutants on biomaterial and are characterized by high long-term stability (31 and 25 days). Toxic exposure leads to a reduction in the period of stable functioning of biosensors by almost half, which is associated with irreversible changes in the conformation of the active centers of microbial cell enzymes (accumulation of toxic effects). This should be taken into account in future studies when preparing a standardized method for determining integral toxicity. The relative standard deviation calculated to characterize operational stability does not exceed 15% in all cases and satisfies the criterion for using biosensors. However, it is worth noting that MFC showed the lowest convergence of results, which limits its use.

### 3.3. Quantitative Assessment of the Toxic Effects of Pollutants on the Bacteria G. oxydans as Part of Biosensors with Different Types of Signal Recording

**Figure 4:**
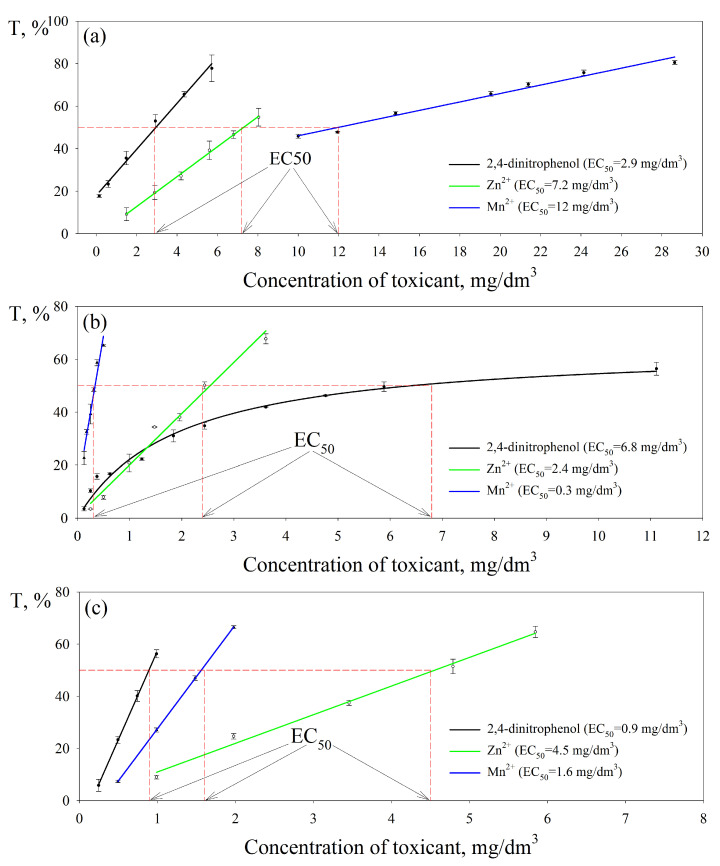
Dependence of the toxicity index of the oxidative activity of bacteria *G. oxydans* on the concentration of toxicants: (**a**) biosensor based on the oxygen electrode; (**b**) mediator type biosensor; (**c**) MFC based biosensor.

To form an effective biosensor for determining integral toxicity, it is necessary to quantify the toxic effect of individual substances on the metabolic activity of microbial cells. To determine the negative impact on the bacteria *G. oxydans*, organic substances that are not oxidized by these bacteria (Figure 3) and are used in the production of polymers as structural components or polymerization catalysts (phenol, 2,4-dinitrophenol, salicylic acid) were used as model toxicants). In addition, a number of heavy metal ions were used (Fe^3+^, Cd^2+^, Mn^2+^, Cr^3+,^ and Zn^2+^), the toxic effects of which have been studied in other bioassay methods [33,34,35,36]. A solution of an organic substance or a heavy metal salt was added to the microbial cells in a cuvette, a stationary current/potential value was waited for (exposure time of at least 5 min) and after adding an aliquot of the substrate, the response of the biosensor was recorded. Thus, under conditions of incubation of microbial cells in a medium with a toxicant, the sensitivity of bacteria was assessed by the value of T, calculated by Formula (3). The inhibition curves of the oxidative activity of *G. oxydans* bacteria as part of different types of biosensors by Mn^2+^, Zn^2+^ ions, and 2,4-dinitrophenol are presented in Figure 4.

The intensity of the inhibitory effect of toxicants in various bioassay methods was assessed by comparing the values of the half-maximal effective concentration EC_50_ of the pollutant, which was determined with a 50% decrease in the oxidative activity of bacteria. Based on the obtained dependences of the index on the concentration of Mn^2+^ ions, it can be noted that the highest EC_50_ value was obtained using a biosystem based on an oxygen electrode and is 12 mg/dm^3^, which indicated the least sensitivity of *G. oxydans* bacteria in this type of sensor to the presence in the environment Mn^2+^ ions. The EC_50_ values of 2,4-dinitrophenol and Zn^2+^ ions were determined using different types of biosensors, comparable to each other, and are values of the same order.

Inhibition curves on the oxidative activity of *G. oxydans* bacteria as part of different electrochemical systems were constructed for all model toxicants studied. The EC_50_ values of inhibitors of the oxidative activity of biorecognition elements of biosensors, presented in Table 2, were compared with the results characterizing the sensitivity of known electrochemical systems [14,16,17,19,37,38,39,40,41] and test objects used in standard methods [33,34,35,36] to the negative effects of the studied toxicants.

The bacteria *G. oxydans* exhibit high sensitivity to the toxic effects of pollutants of an organic and inorganic nature, being part of electrochemical installations with different principles of signal recording. A biosensor based on an oxygen electrode and *G. oxydans* bacteria is more sensitive to Zn^2+^ ions in comparison with analogues [16,37]. However, the EC_50_ of toxicants such as Cd^2+^ ions and phenol, in the case of an oxygen biosensor, is orders of magnitude lower than that of a mediator sensor. The system “*G. oxydans* bacteria–ferrocene–graphite-paste electrode” is superior in sensitivity compared to biosensors formed using an oxygen electrode and MFC. The mediator biosensor is characterized by a greater sensitivity to the presence of heavy metals in the environment (to Cr^3+^ and Mn^2+^ ions at the level of maximum permissible concentrations for water bodies of drinking and cultural water use), which is observed when compared with similar electrochemical systems and biotesting methods, described in the literature [14,16,17,37,40,41]. Using MFC, the EC_50_ of phenol, 2,4-dinitrophenol, and Mn^2+^, Zn^2+^, and Cd^2+^ ions were determined at a level not inferior to other types of biosensors based on the bacteria *G. oxydans* and known prototypes in terms of sensitivity [19,38,39]. This MFC configuration is promising for determining integral toxicity due to its high sensitivity, but to obtain consistent results, it is necessary to use a biocatalyst of the same activity, which can be achieved by immobilizing microbial cells in the anode space.

Despite the low EC_50_ values of the model toxicants obtained using different types of biosensors based on the bacteria *G. oxydans*, standard test objects (*D. magna* [34] and *L. minor* [35,36]) are superior to biosensor analyzers in sensitivity to the toxic effect of heavy metals. However, the use of electrochemical systems based on microbial cells remains an urgent task due to their portability, rapidity, and the possibility of repeated use of the test object for analysis. This indicates the promise of using a mediator biosensor based on the bacteria *G. oxydans* to determine integral toxicity.

### 3.4. Toxicity Analysis of Polymer Samples

**Figure 5:**
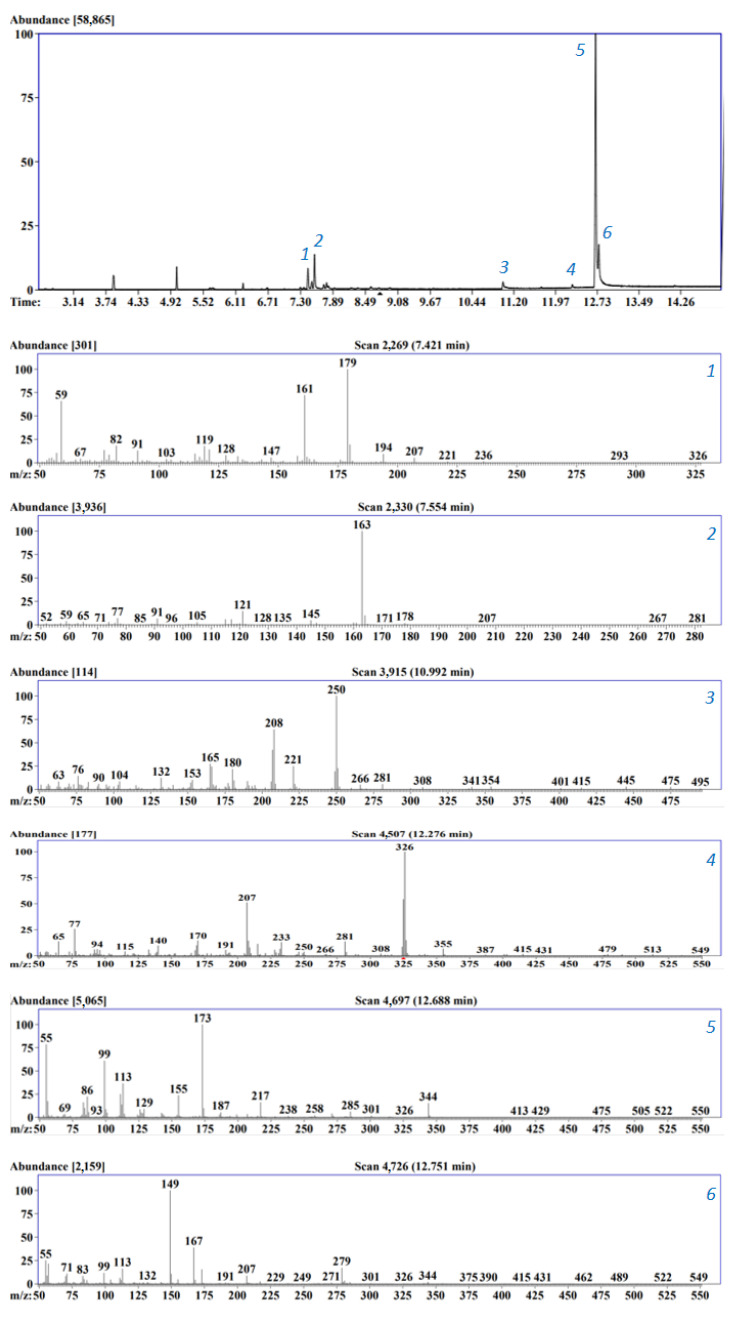
Chromatogram of the phone case sample extract and mass spectra of individual substances separated in a chromatographic column (1—α,α′-dihydroxy-1,3-diisopropylbenzene; 2—4-(1-hydroxy-1-methylethyl)acetophenone; 3—diphenylmethane-4,4′-diisocyanate; 4—triphenyl phosphate; 5—polyethylene adipate; 6—bis (2-ethylhexyl) phthalate).

For the analysis of integral toxicity, six samples of consumer goods made of polymer materials were taken. The gas chromatography-mass spectrometry (GC-MS) method was used to identify the presence of individual toxic substances in their composition. Sample extracts were prepared using acetonitrile since this extractant did not dissolve the polymers under study and is effective in extracting chemical compounds of polar and non-polar nature (phthalates, pesticides) [42,43]. Therefore, as a result of analyzing the extract of the “phone case” sample, a chromatogram was obtained and mass spectra of 6 constituent components of the studied material were identified using the NIST14 library with a score of ≥ 60% (Figure 5).

Unidentified peaks corresponded to contaminants migrating into the sample as a result of erosion of the chromatographic column sorbent (*m*/*z* 73, 207, 281, 355) [44]. In the case of separation of the extract of the “medical gloves” and “pouring gloves” samples, the largest number of peaks (more than 40) were identified, which made it possible to identify only four unique chemical compounds. This is explained by the complex formulation of polymer materials, which includes various additives and fillers, which, during the production and storage of the polymer, can be converted into products of an unknown chemical structure with a specific effect on the body. Thus, using the GC-MS method, substances used as plasticizers and stabilizers in the production of polymer materials, giving them characteristic properties (diphenylmethane-4,4′-diisocyanate, triphenyl phosphate, polyethylene adipate, dimethyl phthalate, bis (2-ethylhexyl) phthalate), and isomers of meso- and terephthalic acids (4-(1-hydroxy-1-methylethyl)acetophenone, α,α′-dihydroxy-1,3-diisopropylbenzene) (Table 3).

The presence of phthalates and isocyanates with proven toxic effects on humans [45,46] and test organisms [47] in the analyzed samples indicates a potential manifestation of the inhibitory effect of the materials under study on the reactions of the test objects used.

Aqueous extracts of samples of industrially produced goods made from polymeric materials were studied for integral toxicity using biosensors with different types of signal recording based on the bacteria *G. oxydans* and standard biotesting methods (test objects: biosensor “Ecolum”, *L. minor*, and cattle sperm). To determine toxicity using the formed electrochemical systems, the response of the biosensor to glucose was assessed (the concentration corresponded to the middle of the linear dependence of the sensor signal on glucose content) in the absence and presence of the test sample (the minimum possible dilution of the test aqueous extract in a measuring cuvette with 30 mM of Na-phosphate buffer with a pH of 6.0 in a ratio of 1:3). T was calculated using Formula (3), taking into account dilution. The toxicity of the sample was recorded when T exceeded 50%. The summary results of testing samples of industrially produced goods for toxicity are presented in Table 4.

A correlation was established for samples No. 2 (toxic) and No. 6 (non-toxic) using biosensors and standard biotesting methods. Dimethyl phthalate in sample No. 6 was not a substance of increased toxicity (Table 3) [48], which is confirmed by the absence of a negative effect on test organisms. However, highly toxic diphenylmethane-4,4′-diisocyanate [49], and components of unknown structure in sample No. 2, led to a significant decrease in the oxidative activity of *G. oxydans* bacteria, the luminescence of the “Ecolum” test system, and the motility of cattle sperm.

The different levels of reaction inhibition in the study of samples No. 1–5 are associated with the unequal sensitivity of the test objects to the toxicants contained. Thus, a higher sensitivity to toxic effects is observed in unicellular organisms compared to duckweed, which is explained by the reduced influence of volatile pollutants on L. minor due to their migration into the air over a long period of analysis (7 days). From the toxicity results of samples No. 3 and No. 4, it can be noted that the bacteria *G. oxydans* were more sensitive to the toxic effects of bis (2-ethylhexyl) phthalate than the “Ecolum” biosensor and sperm.

The sensitivity of the mediator biosensor turned out to be comparable to a biosensor based on MFC, and higher than the sensitivity of the electrochemical system based on an oxygen electrode. This is consistent with the hypothesis about the transport of electrons by a mediator from different parts of the ETC of a bacterial cell, which contributes to the emergence of a specific toxic effect in microorganisms within electrochemical systems of various types due to disruption of biochemical processes.

## 4. Conclusions

Thus, the use of electrochemical systems with different types of signal recording based on the bacteria *G. oxydans* is a promising biotesting method that allows quantitative assessment of the integral toxicity of various materials. The advantage of such microbial sensors is the early warning of the toxic effects of industrially produced products on human health. The rapidity and low cost of these types of biosensors will complement traditional methods of toxicological analysis with sophisticated analytical equipment and comprehensively assess the safety of the materials under study.

The developed biosensors using an oxygen electrode, MFC, and a mediator-type graphite-paste electrode based on the bacteria *G. oxydans* are comparable in sensitivity to the commercial biosensor “Ecolum” and standard biotesting methods, which allows them to be used to assess the toxicity index of consumer goods.

## Figures and Tables

**Table 1:** Main characteristics of biosensors based on bacteria *G. oxydans* for determining toxicity.

Characteristics/Biosensor Type		Biosensor Based on Oxygen Electrode	Mediator- Type Biosensor	Biosensor Based on MFC
K′, mmol/dm^3^		1.8 ± 0.2	16 ± 1	– ^1^
V_max_		0.34 ± 0.01 mgO_2_/(dm^3^ × s)	1.18 ± 0.04 µA	–
Sensitivity factor, 10^−3^		119 ± 3 mgO_2_/(mmol × s)	45 ± 2 µA × dm^3^/mmol	–
The lower limit of glucose detection, mmol/dm^3^		0.01	0.06	–
Operational stability ^2^, %	in the absence of toxicant	6.8	5.6	12.1
	in the presence of Zn^2+^ (EC_50_)	7.4	6.5	14.5
Long-term stability ^3^, days	in the absence of toxicant	31	25	
	in the presence of Zn^2+^ (EC_50_)	18	14	
Single analysis time, min		6–8	5–7	60–80

Note: ^1^ not defined; ^2^ relative standard deviation of 15 biosensor responses to glucose; ^3^ duration of sensor operation without replacing the sensitive element until the oxidative activity of the biomaterial decreases by 50% of the maximum.

**Table 2:** EC_50_ values of model toxicants.

Test Object and Type of Biosensor/Toxicant	EC_50_, mg/dm^3^									Analogue
	Salicylic Acid	TCA	Phenol	2,4-Dinitrophenol	Cd^2+^	Fe^3+^	Cr^3+^	Zn^2+^	Mn^2+^	
Biosensor based on oxygen electrode/*G. oxydans*	>200	>200	>200 [14]	2.9	>200 [14]	16.5	13.9	7.2	12	This study
Mediator biosensor (ferrocene)/*G. oxydans*	19.0	15.7	17.5	6.8	1.6	7.8	0.8	2.4	0.3	This study
Biosensor based on MFC (2,6-DCPIP)/suspension *G. oxydans*	– ^1^	–	24.2	0.9	1.2	–	–	4.5	1.6	This study
Mediator biosensor (ferrocene)/*P. yeei*	ND ^2^	ND	9.9	ND	18.2	ND	ND	47.5	ND	[16]
Biosensor based on oxygen electrode/immobilized *E. coli*	ND	ND	112	ND	11.2	ND	ND	ND	ND	[14]
Mediator biosensor (menadione and potassium hexacyanoferrate (III))/*S.cerevisiae*, *E. coli*	ND	ND	44.5	ND	13.9	ND	ND	ND	ND	[17]
Mediator biosensor (potassium ferrocyanide)/*E. coli* suspension	ND	ND	ND	ND	3.7	>200	10.7	26.7	ND	[37]
MFC (potassium ferricyanide)/Anaerobic sludge	ND	ND	ND	ND	2 (EC_9.29_)	ND	2 (EC_1.95_)	2 (EC_8_)	ND	[19]
MFC HATOX-2000/Activated sludge	ND	ND	ND	ND	ND	0.3 (EC_17_)	ND	1.0 (EC_27_)	1.0 (EC_28_)	[38]
Tox-Alert/*Vibrio fischeri*	43,100	ND	7990	34,690	ND	ND	ND	ND	ND	[39]
Microtox/*Vibrio fischeri*	ND	ND	15.1	ND	40.8	ND	ND	14.5	ND	[40,41]
*Vibrio* sp. MM1	ND	ND	ND	ND	14.54	ND	ND	0.97	ND	[33]
*Daphnia magna*	ND	ND	ND	ND	0.0036	0.002	0.13	0.72	0.0093	[34]
*Lemna minor*	ND	ND	ND	ND	0.33	186.8	11.1; 240,4	0.9;131	ND	[35,36]

^1^ not defined; ^2^ no data.

**Table 3:** Samples of industrially produced goods made from polymeric materials.

No.	Sample	Material	Identified Substances Using GC-MS Method	
			CAS Number	Name
1	Case for phone	Silicone	1999-85-5	α,α′-dihydroxy-1,3-diisopropylbenzene
			54549-72-3	4-(1-hydroxy-1-methylethyl)acetophenone
			101-68-8	diphenylmethane-4,4′-diisocyanate
			115-86-6	triphenyl phosphate
			24938-37-2	polyethylene adipate
			117-81-7	bis (2-ethylhexyl) phthalate
2	Dousing gloves Bottle for water	Polyvinylchloride, polyurethane, textiles	101-68-8	diphenylmethane-4,4′-diisocyanate
			24938-37-2	polyethylene adipate
3	Food container	Polyethyleneterephthalate	117-81-7	bis (2-ethylhexyl) phthalate
4	Medical gloves	Polypropylene	28813-61-8	2-nonadecanone 2,4-dinitrophenylhydrazine
			117-81-7	bis (2-ethylhexyl) phthalate
5	Dousing gloves Bottle for water	Latex	54549-72-3	4-(1-hydroxy-1-methylethyl)acetophenone
			131-11-3	dimethyl phthalate
6	Food container	Polypropylene, silicone	131-11-3	dimethyl phthalate

**Table 4:** Results of integral toxicity of the studied samples (*n* = 5; *p* = 0.95).

No	Sample/Biotest Method	Toxicity Index T, %				Lesser Duckweed *L. minor* ^3^/Yield Inhibition Index I_y_, %	Cattle Sperm (AT-05 Device) ^4^/Motility Index I_t_, %
		Biosensor Based on Oxygen Electrode ^1^	Mediator Biosensor ^1^	Biosensor Based on MFC ^1^	Biosensor “Ecolum” (Device Biotox-10M) ^2^		
1	Casef or phone	64 ± 6	77 ± 1	72 ± 10	43 ± 5	4 ± 1	65.2
2	Dousing gloves	63 ± 3	82.72 ± 0.07	85 ± 8	100 ± 1	22 ± 4	43.6
3	Bottle for water	34 ± 3	74.51 ± 0.04	61 ± 7	0	10 ± 2	108.4
4	Food container	42 ± 4	72.1 ± 0.4	58 ± 7	0	8 ± 2	99.5
5	Medical gloves	38 ± 3	47 ± 4	44 ± 6	75 ± 10	2.4 ± 0.8	100.7
6	Baby bottle with pacifier	0	7 ± 1	10 ± 4	0	0	105.6

^1^ <50%—non-toxic; >50%—toxic. ^2^ <20 %—non-toxic; 20–50%—toxic; >50%—highly toxic. ^3^ <20%—non-toxic; >20%—toxic. ^4^ 70% < I_t_ < 120%—non-toxic; <70% и >120%—toxic.

## Data Availability

Data are contained within the article.
